# Population-based *BRCA* germline mutation screening in the Han Chinese identifies individuals at risk of *BRCA* mutation-related cancer: experience from a clinical diagnostic center from greater Shanghai area

**DOI:** 10.1186/s12885-024-12089-w

**Published:** 2024-04-02

**Authors:** Zhiyuan Wu, Qingyun Zhang, Yiting Jin, Xinju Zhang, Yanli Chen, Can Yang, Xuemei Tang, Haowen Jiang, Xiaoyi Wang, Xinli Zhou, Feng Yu, Bing Wang, Ming Guan

**Affiliations:** 1 Department of Laboratory Medicine, Huashan Hospital, Fudan University, 200040 Shanghai, China; 2 Central Laboratory, Huashan Hospital, Fudan University, 200040 Shanghai, China; 3 Department of General Surgery, Huashan Hospital, Fudan University, 200040 Shanghai, China; 4 Department of Urology, Huashan Hospital, Fudan University, 200040 Shanghai, China; 5 Department of Pancreatic Surgery, Huashan Hospital, Fudan University, 200040 Shanghai, China; 6 Department of Oncology, Huashan Hospital, Fudan University, 200040 Shanghai, China; 7 Health Management Center, Huashan Hospital, Fudan University, 200040 Shanghai, China

**Keywords:** *BRCA* mutation related-cancer, Germline mutation, Population screening

## Abstract

**Background:**

Deleterious *BRCA1*/*2* (*BRCA*) mutation raises the risk for *BRCA* mutation-related malignancies, including breast, ovarian, prostate, and pancreatic cancer. Germline variation of *BRCA* exhibits substantial ethnical diversity. However, there is limited research on the Chinese Han population, constraining the development of strategies for *BRCA* mutation screening in this large ethnic group.

**Methods:**

We profile the *BRCA* mutational spectrum, including single nucleotide variation, insertion/deletion, and large genomic rearrangements in 2,080 apparently healthy Chinese Han individuals and 522 patients with *BRCA* mutation-related cancer, to determine the *BRCA* genetic background of the Chinese Han population, especially of the East Han. Incident cancer events were monitored in 1,005 participants from the healthy group, comprising 11 *BRCA* pathogenic/likely pathogenic (PLP) variant carriers and 994 PLP-free individuals, including 3 LGR carriers.

**Results:**

Healthy Chinese Han individuals demonstrated a distinct *BRCA* mutational spectrum compared to cancer patients, with a 0.53% (1 in 189) prevalence of pathogenic/likely pathogenic (PLP) variant, alongside a 3 in 2,080 occurrence of LGR. *BRCA1* c. 5470_5477del demonstrated high prevalence (0.44%) in the North Han Chinese and penetrance for breast cancer. None of the 3 LGR carriers developed cancer during the follow-up. We calculated a relative risk of 135.55 (95% CI 25.07 to 732.88) for the development of *BRCA* mutation-related cancers in the *BRCA* PLP variant carriers (mean age 42.91 years, median follow-up 10 months) compared to PLP-free individuals (mean age 48.47 years, median follow-up 16 months).

**Conclusion:**

The unique *BRCA* mutational profile in the Chinese Han highlights the potential for standardized population-based *BRCA* variant screening to enhance *BRCA* mutation-related cancer prevention and treatment.

**Supplementary Information:**

The online version contains supplementary material available at 10.1186/s12885-024-12089-w.

## Key message of article

There is significant ethnical diversity in the prevalence and spectrum of *BRCA* germline variants. While previous studies of regional and preliminary national *BRCA* mutation screening have contributed to our knowledge of *BRCA* germline mutation in China, our research has unveiled a distinctive mutational profile in the Han Chinese across major regions of the country, representing 20% of the world’s population. It also demonstrated the potential of *BRCA* mutation screening in the general healthy population for identifying individuals at higher risk of *BRCA* mutation-related cancer, a risk often overlooked by family history-based screening strategies. These findings offer initial insights into the potential benefits of population-based screening for preventing *BRCA* mutation-related cancer in the Chinese Han. Further investigation is warranted, including multi-center, long-term prospective trials, cost-effectiveness analysis, and psychosomatic medical research.

**Supplementary Information:**

The online version contains supplementary material available at 10.1186/s12885-024-12089-w.

## Introduction

Deleterious germline variants of BReast CAncer susceptibility genes *BRCA1* and *BRCA2* (*BRCA)* significantly increase the risk of developing “*BRCA* mutation”-related tumors, including breast, ovarian, pancreatic, and prostate cancer [1]. Screening for these variants in those with a family cancer history has enhanced the early prevention and intervention among high-risk individuals [2, 3].

Large-scale genome databases have expanded our understanding of *BRCA*’s genetic background in the major populations [4], highlighting ethnic diversity in both the prevalence and mutational spectrum of germline *BRCA* variation across Caucasians, Ashkenazi Jews, Hispanics, African Americans, and Asian [5, 6]. In addition, it has brought to light the surprising observation that population-based screening can identify nearly twice as many deleterious variant carriers compared to conventional family history-based screening [7, 8].

In the last five years, there have been over 40 published studies profiling the mutational spectrum in patients with *BRCA* mutation-related cancers in China [9–13]. While some of them revealed the mutational landscape in the healthy controls of case-control studies for breast cancer [9, 14] and ovarian cancer [10], investigations into the prevalence of mutations in the major population (Chinese Han) and the subsequent research on whether variant screening can yield benefits remains limited due to the extensive geographical landscape of China and the significant genetic diversity within the Han ethnic group [15]. Regional studies have documented varying prevalence of single nucleotide variations (SNVs) and small insertion and deletion events (InDels) in areas like Taiwan [16] and Macau [17]. There was also nationwide variant screening conducted, but the participants predominately originates from the North Han and Lingnan Han [18]. Besides, large genomic rearrangements (LGRs), another contributor to the silence of *BRCA* function, have been less reported in this population. It remains uncertain whether broadening *BRCA* screening in this demographic offers more benefits in identifying high-risk individuals [19, 20]. These gaps in our knowledge of *BR*CA variants’ genomic and functional aspects have impeded the establishment and standardization of *BRCA* mutation screening strategy for the Chinese Han population, which constitutes over 20% of the global population.

In this descriptive study, we integrated next-generation sequencing (NGS) data of *BRCA1* and *BRCA2* exons from 2,080 apparently healthy individuals and 522 patients with *BRCA* mutation-related cancer, to reveal the unique genetic pattern of deleterious *BRCA* variants, including SNVs, InDels, and LGRs, in the general Chinese Han population, with a special focus in the East Han, which account for 25% population of the Chinese Han population. Additionally, with clinical follow-up data spanning up to 24 months in the healthy population, we demonstrate that *BRCA* germline mutation screening can aid in the risk stratification and early detection of *BRCA* mutation-related cancer in the apparently healthy Chinese Han population.

## Participants and methods

### Apparently healthy population and patients with *BRCA* mutation related-cancer

From June 2021 to February 2023, 2,080 apparently healthy participants who denied either a personal or family history of cancer were enrolled from the health management center of Huashan Hospital, Fudan University. All the participants were over 18 years old, and their medical records were blindly reviewed by two physicians to confirm the tumor-free status at enrollment. Besides, 121 patients with triple-negative breast cancer (TNBC), 181 with metastatic castration-resistant prostate cancer (mCRPC), 215 with pancreatic ductal adenocarcinoma (PDAC), and 5 with high-grade ovarian cancer (HGOC) seen in Huashan Hospital, Fudan University were enrolled as the *BRCA* mutation related-cancer group. All the cancer patients were enrolled to undergo *BRCA* mutation screening with the aim of formulating surgery and chemo-/radio-therapy strategies guided by their genotypes [21]. The cancer diagnosis were established based on blind review of biopsy or mastectomy slides by 2 certificated pathologists, in accordance to the World Health Organization tumour classification blue book [22–25]. The Han ethnicity and place of birth were confirmed in the electronic healthcare registration system. Written informed consent was received from all participants. In compliance with the Helsinki Declaration of 1975, as revised in 1996, this study was approved by the Institutional Review Board of Huashan Hospital of Fudan University (2023 − 812).

### Germline mutation profiling of *BRCA1* and *BRCA2* by next generation sequencing

Genomic DNA was extracted from ethylenediaminetetraacetic acid anti-coagulated blood using the QIAamp DNA blood mini kit (Qiagen, #51,104). Sequencing library was construction with the *BRCA1* and *BRCA2* gene mutation detection V2 kit (Amoy Diagnostics, #8.06.0092) and sequenced using the MiSeqDx system (Illumina Inc, CA) with a minimum coverage of 200×, uniformity of 95%, and Q30 for over 85% bases.

The germline mutation was called and filtered using the commercial software SSBC-VarScanv1.1.0 developed by Amoy Diagnostics (Xiamen, China). All candidate SNVs or InDels were hard filtered and further confirmed in Integrative Genomics Viewer (IGV). The germline variants in *BRCA1* (MANE NM_007294.4) and *BRCA2* (MANE NM_000059.4) were classified into five categories, including benign, likely benign, variants of uncertain significance, likely pathogenic, and pathogenic following the American College of Medical Genetics (ACMG) guideline (for detailed variant classification protocol, refer to Supplementary File 1 and 2) [26]. *BRCA* databases, including BIC, ClinVar, BRCA Exchange, and LOVD3.0 were used for the population comparative analysis.

### Detection of large genomic rearrangements and confirmation by multiplex ligation probe amplification (MLPA)

The germline copy number variation (CNV) was identified by the AmpliconCnvCaller software from Amoy Diagnostics. Samples with significant CNV in two or more regions of one gene were considered as candidates harboring *BRCA* LGRs and subjected to SALSA MLPA assays (MRC Holland, #P002 for *BRCA1* and #P090 for *BRCA2*) on a PRISM 3500 DNA analyzer (Applied Biosystems, MA) and further validated by the independent kits (MRC Holland, #P087 for *BRCA1* and #P077 for *BRCA2*).

### Follow-up of the apparently healthy participants

The apparently healthy participants received detailed *BRCA* mutation test results through post-test counseling. Those with *BRCA* pathogenic/likely pathogenic (PLP) variants received guidance from the clinical oncologist on self-examination and health follow-ups. From June 2021 to June 2023, 1,005 out of the 2,080 healthy individuals visited to the health management center every 6 to 14 months for tumor risk screening, which included mammography/MRI, breast physical examination (for breast cancer risk), transvaginal ultrasound and CA125 (for ovarian cancer risk), abdominal CT/MRI, CA199 (for pancreatic cancer risk, imaging test was only performed in the individuals with PLP variant), and digital rectal examination, prostate-specific antigen (PSA) (for prostate cancer risk). Over the 24 months during project period, 412 individuals underwent one examination, 375 individuals underwent two, and 218 individuals underwent three follow-ups.

### Association for clinical genomic science (ACGS) classification and computational scoring of variants of unknown significance (VUS)

The VUS obtained by the ACMG criteria were further classified into six categories of pathogenicity: hot, warm, tepid, cool, cold, and ice cold, according to the ACGS classification guideline [27]. Given that the P/LP variants in *BRCA* have emerged in recent human history, rather than deriving from non-human species [28], the evolution conservation-based function prediction tools such as SIFT and polyphen2, were not suitable for annotating missense VUS [29]. Accordingly, these VUS were analyzed for functional pathogenicity with the predictive scoring data from the DNA/protein sequence machine learning-based software iMutant [30], MutaionTaster [31], VEST [32], EVE [33] and REVEL [34].

### Statistical analysis

Statistical analysis and data visualization was performed with R (v4.0.2). Comparison of continuous values was performed using a two-sample *t*-test or Mann-Whitney *U* test if appropriate. Categorical values were compared with Fisher’s exact test. Statistical significance was defined as a two-sided *P* < 0.05.

## Results

### Demographic and genetic background of the participants

The median age for healthy participants was 49.05 (18 to 88) years and 59.77 (19 to 82) years for cancer patients. No gender bias was observed in the healthy group and PDAC patients (Table 1).

**Table 1:** Demographics of Healthy Individuals and *BRCA* Mutation-Related Cancer Carriers

	Male (*n* = )	Age (median) in years	Female (*n* = )	Age (median) in years
**Healthy Individuals**	1198	20–86 (48.95)	882	18–88 (49.20)
**Cancer carriers**	296	31–87 (66.34)	226	19–82 (51.20)
TNBC^a^	0	-	121	19–71 (42.67)
mCRPC^b^	181	31–87(68.45)	0	-
PDAC^c^	115	35–82(63.03)	100	20–82 (61.31)
HGOC^d^	0	-	5	41–71 (55.40)

^a^triple negative breast cancer

^b^castration-resistant prostate cancer

^c^pancreatic ductal adenocarcinoma

^d^high grade ovarian cancer

**Fig. 1:**
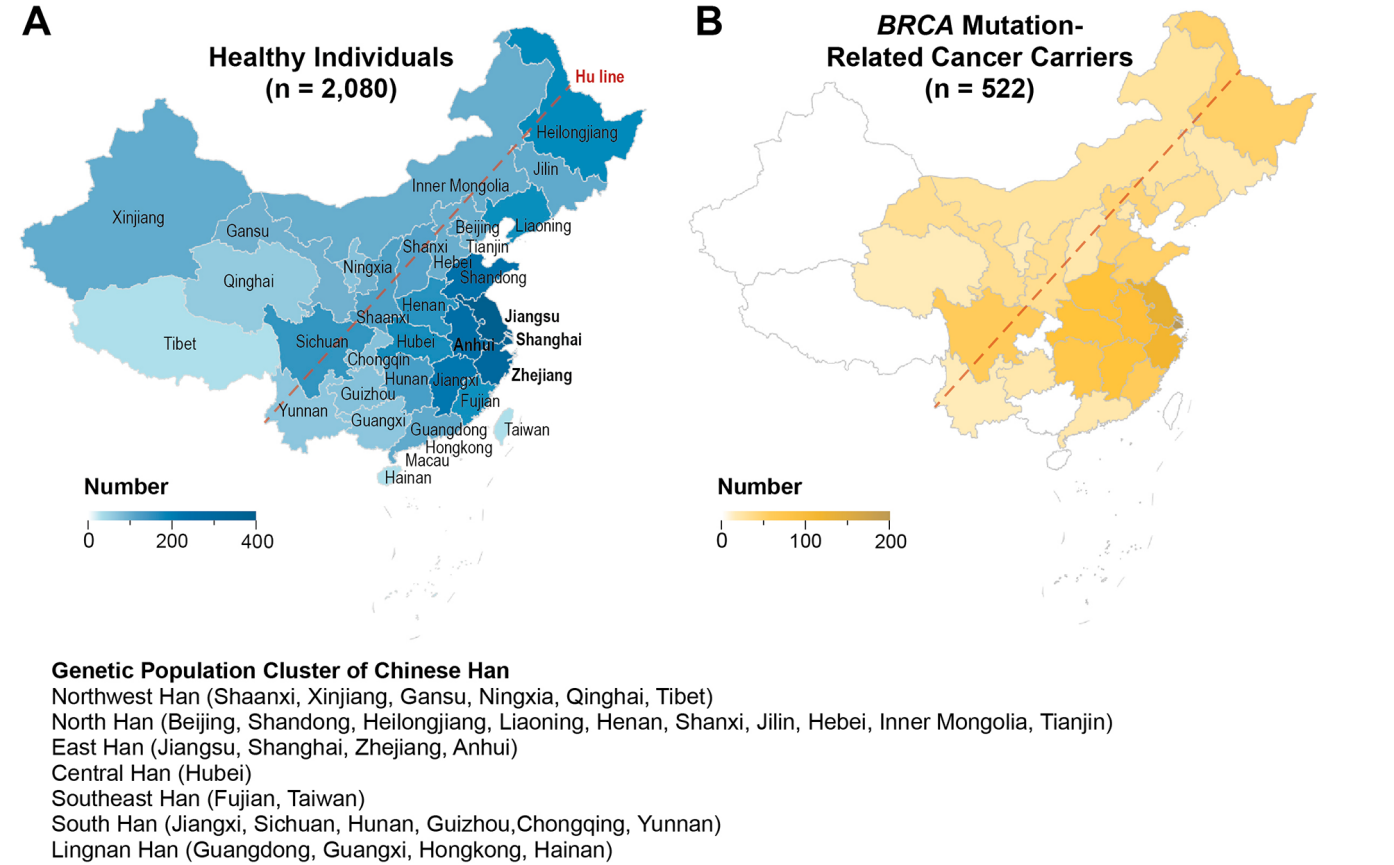
Geographic distribution of the 2,080 healthy individuals and 522 patients with *BRCA* mutation-related cancer. **(A)** Birthplace of the healthy individuals across 33 out the 34 provinces, municipalities and autonomous regions in China (except for Macau SAR), with the majority from east coast and central region (48.71%). **(B)** The major cancer patients are from eastern region of China, represented by the Greater Shanghai area (70.74%)

Among all the 2,080 apparently healthy individuals, there were ten pairs of self-reported first-degree relatives. Three self-reported first-degree relatives were enrolled among all the 522 cancer carriers. The geographic constitution for both the healthy group and cancer cohorts was illustrated in Fig. 1, with the healthy individuals from 33 out of all 34 administrative regions of China, except for Macau Special Administrative Region (SAR), and cancer patients from 25 of these regions. Most of the study population was from the Greater Shanghai area (for healthy group, 17.02% from Shanghai Municipality, 18.7% from Jiangsu Province, 12.99% from Zhejiang Province; for tumor patients, 34.99% from Shanghai Municipality, 21.03% from Jiangsu Province, 14.72% from Zhejiang Province). 97.63% (1960/2020) of the enrolled healthy individuals and 98.45% (509/517) cancer patients are from the region east of the Hu-line, which covered 93% of the population of China. According to the report from ChinaMAP [15], we also subdivided the participants into seven distinguished population clusters, including Northwest Han, North Han, East Han, Central Han, Southeast Han, South Han, and Lingnan Han (Fig. 1). In short, the top three large subpopulation of this study are the East Han (56.40% of healthy individuals and 77.20% of cancer patients), North Han (21.92% of healthy individuals and 9.77% of cancer patients), and South Han (10.14% of healthy individuals and 6.51% of cancer patients). The detailed composition of participants was listed in Supplementary File 3.

### *BRCA* germline variations in the general Chinese Han population and *BRCA* mutation related-cancer cohorts

**Fig. 2:**
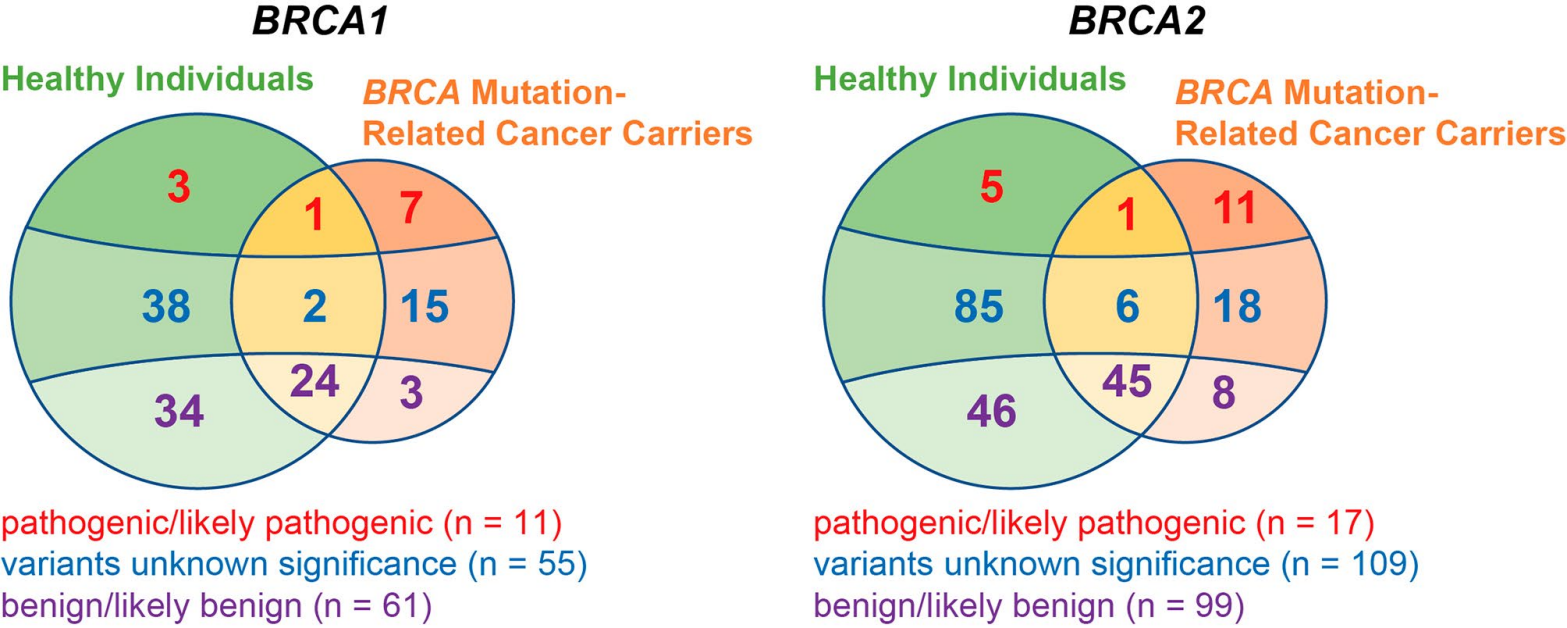
*BRCA* variants identified in the 2,080 healthy individuals and 522 cancer patients. Vien’s diagram illustrates the distribution differences of clinical classified *BRCA* variants between healthy individuals and *BRCA* mutation related-cancer carriers

We gathered 352 distinct germline variants (127 for *BRCA1* and 225 for *BRCA2*) from 2,080 Han Chinese healthy individuals and 522 patients with *BRCA* mutation-related cancer. Among these variations, 211 were specifically identified in healthy individuals and 62 in cancer patients, while 79 variants were present in both two groups (Fig. 2). Over a quarter (134 out of 352) of the variants were recurrent (carriers ≥ 2). Among them, 4 were PLPs, 29 were VUS, and 101 were benign/likely benign (BLB) variants, with 51 healthy cohort-specific and 4 cancer cohort specific variations.

On average, one healthy individual carried 12.09 *BRCA* variants (*BRCA1*: 4.80, *BRCA2*: 7.29), and one cancer patient harbored 12.20 variants (*BRCA1*: 4.97, *BRCA2*: 7.23). No significant difference was observed in the variant burden between the healthy and cancer groups, either for *BRCA1* (*P* = 0.36) or *BRCA2* (*P* = 0.52). Among the healthy population, there was no statistically significant differences in the variant burden among different genders (*P* = 0.72) and among different age groups (*P* = 0.85). There was also no significant regional or cancer species aggregation (*P* = 0.65) of high variation burden. This homogeneity of *BRCA* variant burden across different demographic and pathogenic factors demonstrated a uniform and stable baseline for *BRCA* germline variations in the Chinese Han population.

### *BRCA1/2* pathogenic/likely pathogenic SNV and InDels

Ten PLP variants were identified in the apparently healthy individuals (Table 2) and 20 in the cancer patients (Table 3). There is a 0.53% (11/2080) chance for an individual to harbor the *BRCA* germline PLP variants within our Chinese Han cohort. There was no significant difference between genders [0.33% (4/1198) in males and 0.79% (7/882) in females, *P* = 0.22] and age groups [0.57% (10/1751) for < 60 years old vs. 0.30% (1/329) for ≥ 60 years old, *P* = 1.00] in the incidence of carrying *BRCA* PLP variants.

**Table 2:** Pathogenic/like pathogenic variants in 2,080 Apparently Healthy Han Chinese Individuals

Gene	Exon	cDNA	Amino Acid	Mutation Type	ACMG Classification	Age and Gender of Carrier	Place of Birth (Genetic Cluster)	Developed Cancer during Follow-up?
*BRCA1*	11	c.869del	p.Leu290Tyrfs*8	frameshift deletion	likely pathogenic	29, Male	Jiangsu (East)	No, follow up duration of 20 months
	10	c.2934T > G	p.Tyr978*	nonsense mutation	pathogenic	38, Male	Hunan (South)	No, follow up duration of 24 months
	24	c.5470_5477del	p.Ile1824AspfsTer3	frameshift deletion	pathogenic	51, Female	Shandong (North)	TNBC in the 16th month of follow-up
						30, Female	Henan (North)	No, follow up duration of 5 months
	24	c.5521del	p.Ser1841Valfs*2	frameshift deletion	pathogenic	71, Female	Shandong (North)	No, follow up duration of 19 months
*BRCA2*	11	c.3523 C > T	p.Gln1175Ter	nonsense mutation	pathogenic	33, Female	Zhejiang (East)	TNBC in the 10th month of follow-up
	11	c.3599_3600del	p.Cys1200Ter	nonsense mutation	pathogenic	53, male	Anhui (East)	PDAC in the 9th month of follow-up
	11	c.5682 C > G	p.Tyr1894Ter	nonsense mutation	pathogenic	50, Female	Zhejiang (East)	No, follow up duration of 7 months
	14	c.7409dup	p.Thr2471HisfsTer4	frameshift duplication	pathogenic	48, Male	Jilin (North)	No, follow up duration of 6 months
	21	c.8650del	p.Tyr2884IlefsTer7	frameshift deletion	likely pathogenic	30, Female	Shanghai (East)	No, follow up duration of 7 months
	27	c.9753del	p.Lys3251Asnfs*24	frameshift deletion	pathogenic	39, Female	Jiangsu (East)	No, follow up duration of 21 months

**Table 3:** Pathogenic/like pathogenic variants in 522 Patients with *BRCA* mutation-related cancers

Gene	Exon	cDNA	Amino Acid	Mutation Type	# of Carriers	ACMG Classification	Cancer Type	Gender	Age	Onset of Disease	Place of Birth (Genetic Cluster)
*BRCA1*	7	c.367dup	p.Ser123PhefsTer19	frameshift duplication	1	likely pathogenic	PDAC	Female	52	51	Zhejiang (East)
	11	c.2197_2201del	p.Glu733ThrfsTer5	frameshift deletion	1	pathogenic	TNBC	Female	32	31	Shandong (North)
	11	c.3294del	p.Pro1099Leufs*10	frameshift deletion	1	pathogenic	TNBC	Female	24	22	Anhui (East)
	11	c.3329dup	p.Gln1111Alafs*4	frameshift duplication	1	pathogenic	TNBC	Female	38	36	Jiangsu (East)
	18	c.5138T > C	p.Val1713Ala	nonsynonymous substitution	1	likely pathogenic	TNBC	Female	57	39	Shanghai (East)
	-	c.5194-1del	-	intronic splicing	1	likely pathogenic	TNBC	Female	35	33	Jiangxi (South)
	22	c.5357T > C	p.Leu1786Pro	nonsynonymous substitution	1	likely pathogenic	TNBC	Female	59	58	Shanghai (East)
	24	c.5470_5477del	p.Ile1824AspfsTer3	frameshift deletion	3	pathogenic	TNBC	Female	42	40	Shandong (North)
							TNBC	Female	38	35	Hebei (North)
							TNBC	Female	34	33	Hebei (North)
*BRCA2*	5	c.470_474del	p.Lys157Sfs*24	frameshift deletion	1	pathogenic	TNBC	Female	30	28	Shanghai (East)
	11	c.3847_3848del	p.Val1283Lysfs*2	frameshift deletion	2	pathogenic	PDAC	Male	47	45	Anhui (East)
							PDAC	Male	71	70	Shanghai (East)
	11	c.4630_4631dup	p.Asn1544LysfsTer25	frameshift duplication	1	likely pathogenic	mCRPC	Male	67	63	Jiangsu (East)
	11	c.5073dup	p.Trp1692MetfsTer3	frameshift duplication	1	pathogenic	HGOC	Female	54	47	Shanghai (East)
	11	c.5682 C > G	p.Tyr1894Ter	nonsense mutation	1	pathogenic	TNBC	Female	39	26	Shanghai (East)
	11	c.5980 C > T	p.Gln1994Ter	nonsense mutation	1	pathogenic	mCRPC	Male	74	73	Shanghai (East)
	11	c.6155 C > G	p.Ser2052Ter	nonsense mutation	1	pathogenic	TNBC	Female	46	45	Shanghai (East)
	11	c.6405_6409del	p.Asn2135LysfsTer3	frameshift deletion	1	pathogenic	mCRPC	Male	69	68	Shanghai (East)
	15	c.7480 C > T	p.Arg2494*	nonsense mutation	1	pathogenic	PDAC	Male	64	61	Shandong (North)
	22	c.8941_8942del	p.Glu2981Lysfs*36	frameshift deletion	1	pathogenic	TNBC	Female	29	27	Hubei (Central)
	23	c.9117G > A	p.Pro3039=	synonymous substitution	1	pathogenic	TNBC	Female	35	34	Inner Mongolia (North)
	24	c.9122 C > G	p.Ser3041Ter	nonsense mutation	2	likely pathogenic	TNBC	Female	38	35	Henan (North)
							TNBC	Female	71	71	Henan (North)

The eight healthy individual-specific PLP variants included one frameshift duplication (*BRCA2* c.7409dup), four frameshift deletions (*BRCA1* c.869del, *BRCA1* c.5521del, *BRCA2* c.8650del, *BRCA2* c.9753del), and three nonsense variants (*BRCA1* c.2934T > G, *BRCA2* c.47 C > T, *BRCA2* c.3599_3600del). Any of these variants was not observed in the 1000 genome resource or gnomAD, except for the *BRCA2* c.3599_3600del, which is incorporated in gnomAD with a frequency of 1.09 × 10^− 4^ (1/9,197) in East Asian and 5.29 × 10^− 5^ (3/56,761) in non-Finnish European. Moreover, to our knowledge, the *BRCA1* c.869del, c.2934T > G, c.5521del, and *BRCA2* c.3523 C > T, c.8650del, c.9753del have not been reported by any general population screening study in China. All these eight variants were reported in the ClinVar, BIC, BRCA Exchange, or LOVD database as pathogenic, demonstrating that conducting germline *BRCA* mutation screening in the general Chinese Han population can identify the individuals carrying deleterious variants.

Two nonsense variants, specifically *BRCA1* c.5470_5477del and *BRCA2* c.5682 C > G, were identified in both healthy individuals and cancer patients. Of note, the *BRCA1* c.5470_5477del was present in 5 unrelated individuals − 2 healthy individuals and 3 TNBC patients, all hailing from the North China provinces (Shandong, Hebei, Henan). This variant, previously reported as a founder mutation in the Chinese Han breast cancer patients [35], demonstrated a significant North Han enrichment in both the cancer patients [3/51 (North Han) vs. 0/471 (non-North Han), *P* = 8.84 × 10^− 4^] and healthy individuals [2/456 (North Han) vs. 0/1624 (non-North Han, *P* = 0.048). The *BRCA2* c.5682 C > G was found in 2 unrelated individuals: 1 healthy person and 1 TNBC, both originating from the East Han (Zhejiang and Shanghai). This mutation has been collected in the gnomAD non-Finnish European population, albeit at a low frequency of 1.77 × 10^− 5^ (1/56,574), but it was absent in the other gnomAD population or 1000 genome. Heterozygotes made up all bearers of the PLP variants. Additionally, the recurrent *BRCA2* c.3847_3848del variant was identified exclusively in PDAC patients from the East (Shanghai and Anhui).This variant has also been reported in previous regional studies in the East and Southeast Han (1/2769 unselected breast cancer patient in Zhejiang [36], 1/316 prostate cancer patient in Shanghai [37], and 1/6,314 normal Macan [17]).

**Fig. 3:**
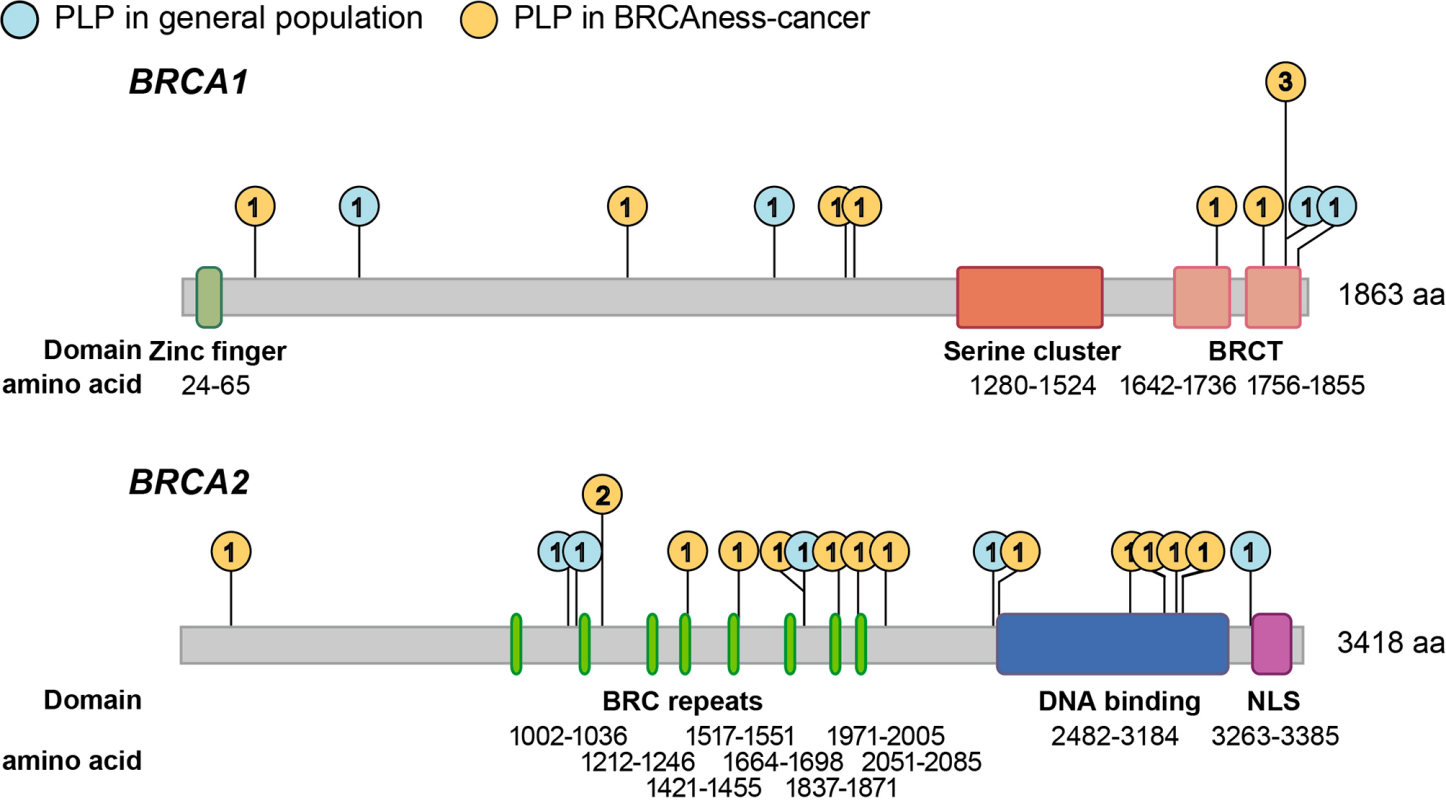
Distribution of PLP variants in the functional domains of *BRCA1* and *BRCA2*. Lollipop plot illustrating the frequency of PLP variants across the functional domain of *BRCA1* [zinc finger, serine cluster, BRCA1 C-terminus (BRCT)] and *BRCA2* [BRC repeats, DNA binding, and nuclear localization signals (NLS)]. The blue circles denote the frequency of PLPs found in the healthy individuals and the orange circles denote PLPs found in *BRCA* mutation related-cancer carriers. PLP variants were clustered in the BRC repeats and DNA binding domain in cancer patients in comparison to the healthy individuals, but not in NLS domain of *BRCA2* and any functional domain of *BRCA1*

Gene-level analysis of variant prevalence revealed no significant enrichment of PLP variants in specific genes when comparing the healthy individuals (4 for *BRCA1*, 6 for *BRCA2*) and cancer patients (8 for *BRCA1*, 12 for *BRCA2*) (Fisher’s Exact *P* = 0.745). The frequency distribution of PLP variants in the gene structures, including UTR, intron, and exon, was similar among healthy individuals and tumor patients for both *BRCA1* and *BRCA2* (Supplementary File 4). However, there was a significant aggregation of PLP variants in the BRC repeats (*P* = 0.019) and DNA binding domain (*P* = 0.015) of *BRCA2* in cancer patients, whereas in healthy individuals, PLP variants were scattered across functional domains (Fig. 3).

Geographically, we observed a significant agglomeration of healthy individuals carrying PLP variants in Yancheng City, with a prevalence of 3.39% (2/59) (Fischer’s Exact, *P* = 0.039). Yancheng City, with a population of 6.69 million, did not exhibit a significantly higher total *BRCA* variant load compared to other cities in China, leading to a unique geographical clustering of PLP variants in this city in the northeastern coastal region of China. We believe that a more extensive screening in local population is necessary to elucidate the interaction between genetics and the environment for cancer risk.

### Case study of recurrent PLP variants’ carriers and incident cancer cases during follow-up

Among all the 2,080 normal individuals, we detected a recurrent pathogenic variant, *BRCA1* c. 5470-5477del, in 2 independent subjects: a 51-year-old female and a 30-year-old female. This variant was also found in three of the 122 TNBC patients, specifically in a 42-year-old female, a 38-year-old female, and a 34-year-old female. Notably, the 51-year-old female carrying *BRCA1* c. 5470-5474del variant developed bilateral breast lesions [Breast imaging-reporting and data system (BI-RAD) 4c, measuring 7 mm × 4 mm for the left and 8 mm × 6 mm for the right] during the follow-up ultrasound examination 16 months after her positive *BRCA* variant screening test. These breast lesions were surgically removed via lumpectomy and confirmed as regional invasive ductal carcinoma (basal-like) by pathology. No other sign of cancer was observed in this patient after the surgical operation.

Additionally, we identified one healthy individual and one TNBC patient sharing the *BRCA2* c. 5682 C > G variant. Over the course of a 7-month follow-up, the 50-year-old female healthy carrier exhibited no clinical manifestation and yielded negative cancer examination results.

In the apparently healthy group, a 70-year-old male carrying *BRCA2* c.3599_3600del nonsense variant was diagnosed with PDAC measuring 36 mm × 34 mm × 21 mm in the head-hook region 9 months after his *BRCA* testing. Furthermore, a 33-year-old female with *BRCA2* c.3523 C > T variant developed invasive mucinous carcinoma in the left breast (measuring 22 mm × 15 mm × 10 mm) during her second annual examination (10 months) after *BRCA* mutation scanning. Additionally, we identified new tumors in two PLP variant-free individuals. One case involved a 59-year-old female diagnosed with left breast TNBC (measuring 24 mm × 15 mm × 15 mm) in the 11th month of her follow-up, and the other case was a 71-year-old male diagnosed with pancreatic body-tail PDAC (measuring 33 mm × 32 mm × 27 mm) in the 24th month of follow-up.

### Similarity and difference of VUS between the general Chinese Han population and cancer cohorts

We also identified 131 VUS in the general Han Chinese population and 41 VUS in the cancer cohorts (Supplementary File 5). Among the 2,080 apparently healthy individuals, we observed 20 recurrent VUS. The most frequently occurring VUS was *BRCA1* c.2726 A > T, found in 8 individuals from major areas of South China, including Shanghai (2 individuals), Zhejiang (2 individuals), Jiangsu (1 individual), Fujian Province (1 individual), and Guangdong Province (1 individual). This VUS was also observed in one patient bearing PDAC, a 71-year-old male from Jiangsu Province.

There is no significant VUS enrichment across the gene structures in the cancer group compared to the healthy group, which differs from the splicing mutations clustering in the tumor group among PLPs (*P* = 0.03). According to the ACGS classification criteria, we observed no significant enrichment of hot/warm variants in the tumor patients compared to the healthy individuals (Fischer’s exact, *P* = 0.96). The reclassification and scoring of VUS by computational prediction tools also revealed that the VUS harbored by PLP variant-free tumor patients and apparently healthy persons did not differ significantly according to the current machine learning algorithm including iMutant (*P* = 0.12), Mutation Taster (*P* = 0.20) and VEST (*P* = 0.81), EVE (*P* = 0.50) and REVEL (*P* = 0.17). This indicates that there should be more extensive research into the pathogenicity of VUS, for example, utilizing the large-scale clinical follow-up data.

In the cancer cohorts, we observed two recurrent VUS in *BRCA2*: c.2186T > C (found in a 69-year-old male from Zhejiang with mCRPC and a 74-year-old female from Shanghai with PDAC) and c.8971 C > T (found in a 28-year-old female from Hebei Province with TNBC and a 52-year-old male from Anhui Province with PDAC). The c. 2186T > C variant also appeared in three healthy individuals (a 76-year-old male from Shanghai, a 43-year-old male from Heilongjiang Province, and a 53-year-old male from Zhejiang), while c.8971 C > T was exclusive to cancer cases. Worth noting is that *BRCA1* c. 3524 C > T was another cancer-specific VUS observed in a PLP-free 61-year-old female patient bearing primary PDAC and TNBC. We also identified *BRCA1* c.548-15G > A in two unrelated healthy individuals, which was previously reported to induce the abnormal transcript splicing [38].

### *BRCA* LGR in the general Chinese Han population and *BRCA* mutation-related cancer cohorts

LGR is another genomic contributor to *BRCA* inactivation beyond SNV and InDels. Using NGS data, we comprehensively analyzed CNV in *BRCA1* and *BRCA2* at amplicon level. After the MLPA experiment, we confirmed the presence of LGR in three healthy individuals, including two relatives with *BRCA2* exon 22 to exon 24 deletion and one subject with *BRCA2* exon 12 to exon 13 duplication. Interestingly, we did not identify any *BRCA* LGR among the 522 cancer patients. These evidence suggest the presence of *BRCA* LGRs in the general Chinese population, although the pathogenicity of these variations needs further validation with longer-term follow-up and broader population cohorts.

### ***BRCA*mutation screening identified individuals at*BRCA*mutation-related cancer risk in the general Chinese Han population**

To assess whether *BRCA* screening can effectively discriminate individuals at elevated risk of *BRCA* mutation-related cancers from the general population, we conducted a prospective follow-up for tumor risk assessment in 1,005 individuals (11 *BRCA* PLP carriers and 994 *BRCA* PLP-free individuals) out of the apparently healthy group after their *BRCA* mutation test.

Throughout the 24-month follow-up period, we identified three new cases of *BRCA* mutation-related cancers (comprising 2 TNBC cases and 1 PDAC case) among the 11 *BRCA* PLP carriers. In the group of 994 *BRCA* PLP-free individuals, there were two new cases (1 TNBC and 1 PDAC). There were no statistically significant differences between the PLP carriers and PLP-free individuals in terms of gender distribution [36.36% (4/11) male (PLP carriers) vs. 56.74% (564/994) male (PLP-free), *P* = 0.23], age [42.91 ± 13.03 years (PLP carriers) vs. 48.47 ± 11.46 years (PLP-free), *P* = 0.11], or follow-up duration [median of 10 months (25th to 75th percentile: 7 to 20 months, PLP carriers) vs. median of 16 month (25th to 75th percentile: 10 to 19 months, PLP-free), *P* = 0.15]. Therefore, the relative risk for developing *BRCA* mutation-related cancer in the exposure to a positive *BRCA* germline mutation test is 135.55 (95% CI 25.07 to 732.88), with an absolute risk increasement = 27.07% (95% CI = 23.24–30.90%).

## Discussion

*BRCA* germline mutation carriers face a high risk for *BRCA* mutation-related cancers. While *BRCA* variant screening effectively aids risk classification and prevention in those with familial history of breast/ovarian cancer [2, 39, 40], the mutational spectrum shifts across ethnicities [5, 6], causing debates about population-wide screening and its implementation [20].

Prior studies have explored the *BRCA* germline variants in the Chinese population, but challenges remain unaddressed: (1) most studies have concentrated on patients already diagnosed with *BRCA* mutation-related cancer [11, 14, 41]; (2) population-based studies on healthy individuals are regionally restricted (Taiwan [16], Macau [17], North China [18]; 3) there has been a lack of post-test follow-up to determine whether screening in the general population identifies high cancer risk individuals. The functional landscape of *BRCA* germline variation in the world’s largest genetic population, the Han Chinese, remains inadequately understood.

This study presents our experience in *BRCA* germline variant screening involving 2,080 apparently healthy population and 522 *BRCA* mutation-related cancer patients. It covered 33 of the 34 administrative regions in China, except Macau SAR, offering a diverse genetic representation of the Chinese Han Population. With a centralized recruitment, testing, and follow-up process, our pipeline ensured consistent and reliable conclusions.

We found an incidence of 0.53% (one in 189) for a Han Chinese to carry germline *BRCA* pathogenic or likely pathogenic variants. By consolidating our findings with those of previous studies in China, such as Dong et al. (0.53%, *n* = 11,386 normal Chinese) [18], Qin et al. (0.38%, *n* = 6,314 normal Macanese) [17], Chain et al. (0.53%, *n* = 1,517 Taiwanese) [16], Liu et al. (1.10%, *n* = 6,434 normal control for breast cancer) [9], Lang et al. (0.38%, *n* = 1,043 normal control for breast cancer) [14], and Li et al. (0.34%, *n* = 1,763 normal control for ovarian cancer), we estimated a 0.52% (95% CI = 0.30–0.84%) prevalence of deleterious *BRCA* mutations in the Chinese Han. This is lower than the established rate in Ashkenazi Jews (2%) ​ [42]​, similar to the American and British populations (0.5%) [43], and slightly higher than other East Asian populations, including Japanese and Korean (0.2%) [44]. The consistent variant frequency across various Chinese studies underscores the stable baseline of *BRCA* germline variations in this demographic. However, we also discovered significant regional differences in the mutational spectrum. For example, the founder mutation *BRCA1* c.5470_5477del is specifically harbored by the North Han in our study (0.44% in healthy individuals and 5.89% in cancer patients), and this variant has not been reported in previous *BRCA* variant screening studies conducted in the south region of China [45–47]. Furthermore, the PLP variants found in our healthy group, including *BRCA1* c.2934, c.5521del, c.869del, and *BRCA2* c.3523 C > T, c.8650del, c.9753del have not been reported in previous screenings of the normal Chinese population [9, 10, 14, 16–18].

Of note, all identified PLP carriers denied a family history of cancer during the pre-test genetic counseling, and there were no serological or radiological indications of tumors. These oversights emphasized the limitations of family history-based screening strategy: it mandates the presence of a family member with cancer diagnosis and a well-documented family history of disease.

The mutational spectrum of PLP variants differs between healthy individuals and cancer patients. Among the 11 *BRCA1* PLPs identified in our study, only one (c. 5470_5477del) was common to both healthy individuals and cancer patients. Similarly, these two groups shared only one of the 17 *BRCA2* PLPs (c. 5682 C > G). However, the presence of these PLPs in healthy individuals does not negate their pathogenicity. Actually, three out of the 11 individuals carrying these PLPs developed *BRCA* mutation-related cancer during follow-up. For instance, *BRCA1* c.5470_5477del showed a relatively high prevalence (0.26%, 3/1151) in the North Han and demonstrated penetrance for TNBC [35]. In addition, more efforts should be encouraged on further categorizing the pathogenicity of VUS, such as the recurrent *BRCA2* c.8971 C > T in the cancer cohort and the potential splicing abnormalities causing *BRCA1* c.548-15G > A in the healthy group. Long-term phenotypic follow-up will provide evidence-based medicine level insight beyond the current machine-learning approach.

In contrast to the aggregation of PLP variants within the functional domains of *BRCA* (*BRCA2* BRC repeat and DNA-binding) in cancer patients [48, 49], we observed a uniformed distribution of PLPs across *BRCA1* and *BRCA2* sequence in the healthy individuals. This supports the hypothesis that *BRCA* pathogenic variants originated relatively recently in human history [28]; however, further disease penetrating restricted the complexity of the variants into a specific genomic region. These findings highlight the necessity of employing NGS for germline mutation screening in *BRCA*.

Apart from SNV and small insertion/deletion, we observed two types of LGRs in three out of the 2,080 healthy individuals. These included two individuals with kinship harboring the same exon 22 to exon 24 deletion in *BRCA2* [50]. Notably, LGRs were not observed in the 522 cancer patients. All three individuals with LGRs have not shown any sign of developing malignancies so far, even after follow-ups at the ages of 52, 55, and 78. This explains the relatively low penetrance of LGR (~ 1%) in *BRCA* mutation-related cancers in China [51, 52], compared to European patients [53, 54]. However, long-term follow-up beyond the 24-month shall be encouraged to elucidate the pathogenicity of these structure variants. It also highlights the need for a sensitive and specific algorithm for LGR calling using NGS data.

Our follow-up on 1,005 healthy Chinese Han individuals showed that those with positive *BRCA* variant tests had significantly increased *BRCA* mutation-related cancer risks (RR = 135.55, 95% CI 25.07 to 732.88) after accounting for potential confounders, including age, gender, and duration of follow-up. While the relatively small sample size in the PLP carrier group might cause overestimation, it highlights the value of *BRCA* mutation screening in the general Chinese Han population.

There is emerging evidence that population-wide screening is a better approach for the prevention of *BRCA* mutation-related cancer since family history-based screening misses a significant portion of individuals carrying the *BRCA* variant [7]. Considering the relatively high prevalence and mutational profile background in this context, the unique “small family” structure within the major Chinese populations, and the widespread culture of “medical stigmatization” in East Asia, we suggest broader *BRCA* variant screening, accompanied by detailed comprehensive genetic counseling.

One limitation of this study is the relatively short follow-up period, which may not adequately reflect the relative risk of cancer development in PLP carriers compared to PLP-free individuals. Additionally, since the participation of follow-up is voluntary other than mandatory, healthy individuals with the PLP-free results from *BRCA* mutation screening may have limited willingness to participate in the follow-up, leading to a 50% follow-up rate in this study. Furthermore, although our study included 2,080 healthy individuals, the regional sampling bias limited our findings primarily to the East Han population, and does not fully represent the situation within the 1.4 billion Chinese Han population. Moreover, While the single-center design enhanced the comparability of the testing and follow-up data, it also introduced sampling bias and other unexpected confounders. Therefore, a multi-center prospective study is encouraged to elucidate the medical benefits of population-based screening of *BRCA* germline variants in the Chinese Han population. Further cost-effectiveness studies comprehending the balance between variant screening and financial expenditure [55, 56], and psychosocial studies [57, 58] on the impact of genetic test results, will facilitate devising the optimal screening strategy in the Chinese Han population.

## Conclusion

By integrating NGS data from 2,080 apparently healthy individuals, we have characterized the genetic landscape of germline *BRCA* variants, including SNVs, small InDels and LGRs, in the Chinese Han, with a special focus on the East Han subpopulation. The mutational spectrums are of significant difference between the healthy individuals and cancer patients. Furthermore, we conducted a short-term follow-up involving 1,005 individuals from the healthy group, confirming that individuals identified as PLP carriers by population-based screening face a significantly elevated risk of developing *BRCA* mutation-related cancer compared to those without PLPs.

Our study highlights the utility of *BRCA* germline variant screening for risk stratification and early cancer detection in the apparently healthy Chinese Han individuals. We advocate for multi-center prospective studies to assess the medical benefits of population-based *BRCA* germline variant screening compared to conventional family history-based screening in the Chinese Han population. Additionally, we anticipate that our research, along with investigations into financial considerations and psychosocial impact of genetic test results, will contribute to the development of an optimal screening strategy for the Chinese Han population.

### Electronic supplementary material

Below is the link to the electronic supplementary material.

Supplementary Material 1

Supplementary Material 2

Supplementary Material 3

Supplementary Material 4

Supplementary Material 5

## Data Availability

The datasets used and/or analysed during the current study are available in the main text and supplementary files of the manuscript.

## Abbreviations

BRCA: *BRCA1*/*2*
SNV: Single nucleotide variation
InDel: Insertion and deletion
LGR: Large genomic rearrangement
NGS: Next-generation sequencing
TNBC: Triple-negative breast cancer
mCRPC: Metastatic castration-resistant prostate cancer
PDAC: Pancreatic ductal adenocarcinoma
HGOC: High-grade ovarian cancer
ACMG: American College of Medical Genetics
CNV: Copy number variation
PLP: Pathogenic/likely pathogenic
ACGS: Association for Clinical Genomic Science
VUS: Variant of unknown significance
BLB: benign/likely benign
BI-RAD: Breast imaging-reporting and data system
RR: Relative risk

## References

[1] Lord CJ, Ashworth A (2016). BRCAness revisited. Nat Rev Cancer.

[2] Paluch-Shimon S, Cardoso F, Sessa C, Balmana J, Cardoso MJ, Gilbert F, Senkus E, Committee EG (2016). Prevention and screening in BRCA mutation carriers and other breast/ovarian hereditary cancer syndromes: ESMO Clinical Practice guidelines for cancer prevention and screening. Ann Oncol.

[3] Daly MB, Pilarski R, Yurgelun MB, Berry MP, Buys SS, Dickson P, Domchek SM, Elkhanany A, Friedman S, Garber JE (2020). NCCN guidelines insights: Genetic/Familial High-Risk Assessment: breast, ovarian, and pancreatic, Version 1.2020. J Natl Compr Canc Netw.

[4] Karczewski KJ, Francioli LC, Tiao G, Cummings BB, Alfoldi J, Wang Q, Collins RL, Laricchia KM, Ganna A, Birnbaum DP (2020). The mutational constraint spectrum quantified from variation in 141,456 humans. Nature.

[5] Rebbeck TR, Friebel TM, Friedman E, Hamann U, Huo D, Kwong A, Olah E, Olopade OI, Solano AR, Teo SH (2018). Mutational spectrum in a worldwide study of 29,700 families with BRCA1 or BRCA2 mutations. Hum Mutat.

[6] Bhaskaran SP, Chandratre K, Gupta H, Zhang L, Wang X, Cui J, Kim YC, Sinha S, Jiang L, Lu B (2019). Germline variation in BRCA1/2 is highly ethnic-specific: evidence from over 30,000 Chinese hereditary breast and ovarian cancer patients. Int J Cancer.

[7] Manchanda R, Sun L, Patel S, Evans O, Wilschut J, De Freitas Lopes AC, Gaba F, Brentnall A, Duffy S, Cui B et al. Economic evaluation of Population-based BRCA1/BRCA2 mutation testing across multiple countries and Health systems. Cancers (Basel) 2020, 12(7). 10.3390/cancers12071929 PMC7409094 32708835

[8] Kemp Z, Turnbull A, Yost S, Seal S, Mahamdallie S, Poyastro-Pearson E, Warren-Perry M, Eccleston A, Tan MM, Teo SH (2019). Evaluation of Cancer-based Criteria for Use in Mainstream BRCA1 and BRCA2 genetic testing in patients with breast Cancer. JAMA Netw Open.

[9] Liu Y, Wang H, Wang X, Liu J, Li J, Wang X, Zhang Y, Bai Z, Zhou Q, Wu Y (2021). Prevalence and reclassification of BRCA1 and BRCA2 variants in a large, unselected Chinese Han breast cancer cohort. J Hematol Oncol.

[10] Li A, Xie R, Zhi Q, Deng Y, Wu Y, Li W, Yang L, Jiao Z, Luo J, Zi Y (2018). BRCA germline mutations in an unselected nationwide cohort of Chinese patients with ovarian cancer and healthy controls. Gynecol Oncol.

[11] Yin L, Wei J, Lu Z, Huang S, Gao H, Chen J, Guo F, Tu M, Xiao B, Xi C (2022). Prevalence of germline sequence variations among patients with pancreatic Cancer in China. JAMA Netw Open.

[12] Zhu Y, Wei Y, Zeng H, Li Y, Ng CF, Zhou F, He C, Sun G, Ni Y, Chiu PKF (2021). Inherited mutations in Chinese men with prostate Cancer. J Natl Compr Canc Netw.

[13] Lei H, Zhang M, Zhang L, Hemminki K, Wang XJ, Chen T (2022). Overview on population screening for carriers with germline BRCA mutation in China. Front Oncol.

[14] Lang GT, Shi JX, Hu X, Zhang CH, Shan L, Song CG, Zhuang ZG, Cao AY, Ling H, Yu KD (2017). The spectrum of BRCA mutations and characteristics of BRCA-associated breast cancers in China: screening of 2,991 patients and 1,043 controls by next-generation sequencing. Int J Cancer.

[15] Cao Y, Li L, Xu M, Feng Z, Sun X, Lu J, Xu Y, Du P, Wang T, Hu R (2020). The ChinaMAP analytics of deep whole genome sequences in 10,588 individuals. Cell Res.

[16] Chian J, Sinha S, Qin Z, Wang SM (2021). BRCA1 and BRCA2 variation in Taiwanese General Population and the Cancer Cohort. Front Mol Biosci.

[17] Qin Z, Kuok CN, Dong H, Jiang L, Zhang L, Guo M, Leong HK, Wang L, Meng G, Wang SM (2021). Can population BRCA screening be applied in non-ashkenazi jewish populations? Experience in Macau population. J Med Genet.

[18] Dong H, Chandratre K, Qin Y, Zhang J, Tian X, Rong C, Wang N, Guo M, Zhao G, Wang SM (2021). Prevalence of BRCA1/BRCA2 pathogenic variation in Chinese Han population. J Med Genet.

[19] Yurgelun MB, Hiller E, Garber JE (2015). Population-wide screening for germline BRCA1 and BRCA2 mutations: too much of a good thing?. J Clin Oncol.

[20] Ficarazzi F, Vecchi M, Ferrari M, Pierotti MA (2021). Towards population-based genetic screenings for breast and ovarian cancer: a comprehensive review from economic evaluations to patient perspectives. Breast.

[21] Daly MB, Pal T, Berry MP, Buys SS, Dickson P, Domchek SM, Elkhanany A, Friedman S, Goggins M, Hutton ML (2021). Genetic/Familial High-Risk Assessment: breast, ovarian, and pancreatic, Version 2.2021, NCCN Clinical Practice guidelines in Oncology. J Natl Compr Canc Netw.

[22] Tan PH, Ellis I, Allison K, Brogi E, Fox SB, Lakhani S, Lazar AJ, Morris EA, Sahin A, Salgado R (2020). The 2019 World Health Organization classification of tumours of the breast. Histopathology.

[23] Humphrey PA, Moch H, Cubilla AL, Ulbright TM, Reuter VE (2016). The 2016 WHO classification of Tumours of the urinary system and male genital organs-Part B: prostate and bladder tumours. Eur Urol.

[24] Nagtegaal ID, Odze RD, Klimstra D, Paradis V, Rugge M, Schirmacher P, Washington KM, Carneiro F, Cree IA, Board WHOCTE (2020). The 2019 WHO classification of tumours of the digestive system. Histopathology.

[25] McCluggage WG, Singh N, Gilks CB (2022). Key changes to the World Health Organization (WHO) classification of female genital tumours introduced in the 5th edition (2020). Histopathology.

[26] Richards S, Aziz N, Bale S, Bick D, Das S, Gastier-Foster J, Grody WW, Hegde M, Lyon E, Spector E (2015). Standards and guidelines for the interpretation of sequence variants: a joint consensus recommendation of the American College of Medical Genetics and Genomics and the Association for Molecular Pathology. Genet Med.

[27] Chen D (2023). [A Chinese interpretation for the ACGS Best Practice guidelines for variant classification in Rare Disease 2020]. Zhonghua Yi Xue Yi Chuan Xue Za Zhi.

[28] Li J, Zhao B, Huang T, Qin Z, Wang SM. Human BRCA pathogenic variants were originated during recent human history. Life Sci Alliance 2022, 5(5). 10.26508/lsa.202101263 PMC8860097 35165121

[29] Poon KS (2021). In silico analysis of BRCA1 and BRCA2 missense variants and the relevance in molecular genetic testing. Sci Rep.

[30] Capriotti E, Fariselli P, Casadio R (2005). I-Mutant2.0: predicting stability changes upon mutation from the protein sequence or structure. Nucleic Acids Res.

[31] Schwarz JM, Rodelsperger C, Schuelke M, Seelow D (2010). MutationTaster evaluates disease-causing potential of sequence alterations. Nat Methods.

[32] Carter H, Douville C, Stenson PD, Cooper DN, Karchin R (2013). Identifying mendelian disease genes with the variant effect scoring tool. BMC Genomics.

[33] Frazer J, Notin P, Dias M, Gomez A, Min JK, Brock K, Gal Y, Marks DS (2021). Disease variant prediction with deep generative models of evolutionary data. Nature.

[34] Ioannidis NM, Rothstein JH, Pejaver V, Middha S, McDonnell SK, Baheti S, Musolf A, Li Q, Holzinger E, Karyadi D (2016). REVEL: an Ensemble Method for Predicting the pathogenicity of rare missense variants. Am J Hum Genet.

[35] Meng H, Yao L, Yuan H, Xu Y, Ouyang T, Li J, Wang T, Fan Z, Fan T, Lin B (2020). BRCA1 c.5470_5477del, a founder mutation in Chinese Han breast cancer patients. Int J Cancer.

[36] Deng M, Chen HH, Zhu X, Luo M, Zhang K, Xu CJ, Hu KM, Cheng P, Zhou JJ, Zheng S (2019). Prevalence and clinical outcomes of germline mutations in BRCA1/2 and PALB2 genes in 2769 unselected breast cancer patients in China. Int J Cancer.

[37] Wei Y, Wu J, Gu W, Qin X, Dai B, Lin G, Gan H, Freedland SJ, Zhu Y, Ye D (2019). Germline DNA repair Gene Mutation Landscape in Chinese prostate Cancer patients. Eur Urol.

[38] Dong Z, Wang Y, Zhang J, Zhu F, Liu Z, Kang Y, Lin M, Shi H (2023). Analyzing the effects of BRCA1/2 variants on mRNA splicing by minigene assay. J Hum Genet.

[39] Valencia OM, Samuel SE, Viscusi RK, Riall TS, Neumayer LA, Aziz H (2017). The role of genetic testing in patients with breast Cancer: a review. JAMA Surg.

[40] Sessa C, Balmaña J, Bober SL, Cardoso MJ, Colombo N, Curigliano G, Domchek SM, Evans DG, Fischerova D, Harbeck N (2023). Risk reduction and screening of cancer in hereditary breast-ovarian cancer syndromes: ESMO Clinical Practice Guideline. Ann Oncol.

[41] Wu X, Wu L, Kong B, Liu J, Yin R, Wen H, Li N, Bu H, Feng Y, Li Q (2017). The First Nationwide Multicenter Prevalence Study of Germline BRCA1 and BRCA2 mutations in Chinese ovarian Cancer patients. Int J Gynecol Cancer.

[42] Hartge P, Struewing JP, Wacholder S, Brody LC, Tucker MA (1999). The prevalence of common BRCA1 and BRCA2 mutations among Ashkenazi jews. Am J Hum Genet.

[43] Manchanda R, Blyuss O, Gaba F, Gordeev VS, Jacobs C, Burnell M, Gan C, Taylor R, Turnbull C, Legood R (2018). Current detection rates and time-to-detection of all identifiable BRCA carriers in the Greater London population. J Med Genet.

[44] Maxwell KN, Domchek SM, Nathanson KL, Robson ME (2016). Population frequency of germline BRCA1/2 mutations. J Clin Oncol.

[45] Wang Q, Wu H, Lan Y, Zhang J, Wu J, Zhang Y, Li L, Liu D, Zhang J (2021). Changing patterns in clinicopathological characteristics of breast Cancer and prevalence of BRCA mutations: analysis in a rural area of Southern China. Int J Gen Med.

[46] Luo Y, Wu H, Huang Q, Rao H, Yu Z, Zhong Z (2022). The features of BRCA1 and BRCA2 germline mutations in Hakka Ovarian Cancer patients: BRCA1 C.536 A > T maybe a founder mutation in this Population. Int J Gen Med.

[47] Shui L, Li X, Peng Y, Tian J, Li S, He D, Li A, Tian B, Li M, Gao H (2021). The germline/somatic DNA damage repair gene mutations modulate the therapeutic response in Chinese patients with advanced pancreatic ductal adenocarcinoma. J Transl Med.

[48] Roy R, Chun J, Powell SN (2011). BRCA1 and BRCA2: different roles in a common pathway of genome protection. Nat Rev Cancer.

[49] Heramb C, Wangensteen T, Grindedal EM, Ariansen SL, Lothe S, Heimdal KR, Maehle L (2018). BRCA1 and BRCA2 mutation spectrum - an update on mutation distribution in a large cancer genetics clinic in Norway. Hered Cancer Clin Pract.

[50] Hua D, Tian Q, Wang X, Bei T, Cui L, Zhang B, Bao C, Bai Y, Zhao X, Yuan P (2022). Next-generation sequencing based detection of BRCA1 and BRCA2 large genomic rearrangements in Chinese cancer patients. Front Oncol.

[51] Cao WM, Zheng YB, Gao Y, Ding XW, Sun Y, Huang Y, Lou CJ, Pan ZW, Peng G, Wang XJ (2019). Comprehensive mutation detection of BRCA1/2 genes reveals large genomic rearrangements contribute to hereditary breast and ovarian cancer in Chinese women. BMC Cancer.

[52] Su L, Zhang J, Meng H, Ouyang T, Li J, Wang T, Fan Z, Fan T, Lin B, Xie Y (2018). Prevalence of BRCA1/2 large genomic rearrangements in Chinese women with sporadic triple-negative or familial breast cancer. Clin Genet.

[53] Riahi A, Chabouni-Bouhamed H, Kharrat M (2017). Prevalence of BRCA1 and BRCA2 large genomic rearrangements in Tunisian high risk breast/ovarian cancer families: implications for genetic testing. Cancer Genet.

[54] Bozsik A, Pocza T, Papp J, Vaszko T, Butz H, Patocs A, Olah E. Complex characterization of Germline large genomic rearrangements of the BRCA1 and BRCA2 genes in high-risk breast Cancer patients-Novel variants from a large National Center. Int J Mol Sci 2020, 21(13). 10.3390/ijms21134650 PMC7370166 32629901

[55] Manchanda R, Legood R, Burnell M, McGuire A, Raikou M, Loggenberg K, Wardle J, Sanderson S, Gessler S, Side L (2015). Cost-effectiveness of population screening for BRCA mutations in Ashkenazi jewish women compared with family history-based testing. J Natl Cancer Inst.

[56] Manchanda R, Patel S, Gordeev VS, Antoniou AC, Smith S, Lee A, Hopper JL, MacInnis RJ, Turnbull C, Ramus SJ (2018). Cost-effectiveness of Population-based BRCA1, BRCA2, RAD51C, RAD51D, BRIP1, PALB2 mutation testing in Unselected General Population women. J Natl Cancer Inst.

[57] Halbert CH, Stopfer JE, McDonald J, Weathers B, Collier A, Troxel AB, Domchek S (2011). Long-term reactions to genetic testing for BRCA1 and BRCA2 mutations: does time heal women’s concerns?. J Clin Oncol.

[58] Watson M, Foster C, Eeles R, Eccles D, Ashley S, Davidson R, Mackay J, Morrison PJ, Hopwood P, Evans DG (2004). Psychosocial impact of breast/ovarian (BRCA1/2) cancer-predictive genetic testing in a UK multi-centre clinical cohort. Br J Cancer.

