# Supplementary material for: Population-based BRCA germline mutation screening in the Han Chinese identifies individuals at risk of BRCA mutation-related cancer: experience from a clinical diagnostic center from greater Shanghai area
Source: BMC Cancer. 2024 Apr 2;24:411. doi: 10.1186/s12885-024-12089-w (PMC10988807; doi:10.1186/s12885-024-12089-w)
Supplement: Supplementary file 1 — Supplementary Material 1 [file 12885_2024_12089_MOESM1_ESM.docx]

**Supplementary File 1**

**Standardized Procedure for Interpretation of *BRCA* Variants within NGS project**

1. **Objective**

Establish standardized procedures for the interpretation of BRCA1/2 gene variants within NGS projects.

1. **Reference Definition**

This process primarily draws upon the ACMG Genetic Variation Classification Standards and Guidelines from 2015 (https://www.gimjournal.org/article/S1098-3600(21)03031-8/fulltext), in conjunction with the ENIGMA BRCA1/2 Mutation Classification Standard from 2017 (https://enigmaconsortium.org/), and the Interpretation of Chinese Expert Consensus on BRCA1/2 Data from 2021 (https://rs.yiigle.com/CN112151202106/1322775.htm).

1. **Procedure**
   1. Overview of General Rules
2. The fundamental framework and structure of this protocol align with ACMG Genetic Variation Classification Standards and Guidelines (hereinafter referred to as the “Guidelines”), with minor variations in evidence utilization.
3. The evidence for interpretation of variants is categorized into eight distinct types: population data, computational predictions, functional data, co-segregation data, de novo data, allelic data, other databases, and miscellaneous data. Simultaneously, based on the evidence's strength and directionality, it is classified into seven levels: solitary benign evidence (BA), robust benign evidence (BS), supportive benign evidence (BP), highly robust pathogenic evidence (PVS), robust pathogenic evidence (PS), moderate pathogenic evidence (PM), and corroborative pathogenic evidence (PP) (refer to Appendix A and Appendix B). The collected evidence can be summarized and comprehensively assessed at the corresponding points (refer to Appendix C).
4. In specific applications, certain pieces of evidence may be adjusted, either upwards or downwards, depending on the specific circumstances.
5. Based on the aforementioned guidelines, the entire interpretation process can be divided into the following stages: allele frequency inquiry, variant type analysis, querying public databases, literature review, computational predictions, comprehensive evidence analysis, and final judgment.
6. Genetic diseases associated with the BRCA1/2 gene predominantly encompass breast cancer, ovarian cancer, prostate cancer, and pancreatic cancer. Unless otherwise specified, all patients referred to in this protocol are affected by these diseases.
   1. Interpretation Steps and Precautions
      1. Allele Frequency Inquiry (BA1/BS1/PM2)
7. This step involves verifying the allele frequency of the variant by querying public population databases, primarily including 1000Genomes (http://grch37.ensembl.org/Homo_sapiens/Info/Index), gnomAD and ExAC (https://gnomad.broadinstitute.org/).
8. Depending on the specific allele frequency (AF) within the population, corresponding evidence can be determined: AF ≥ 1% (BA1), AF ≥ 0.1% (BS1), AF < 0.03% (PM2). Please note that both the total number of alleles (AN) and the number of variant alleles (AC) must be taken into account, and the classification criteria are as follows:

| **Allele Frequency** **(AF)** | **AC and AN consideration** | **Evidence** |
| --- | --- | --- |
| ≥ 1% | AC ≥ 2, AN ≥ 100 | BA1 |
| ≥ 0.1% | AN ≥ 15000 | BS1 |
| ≥ 0.5% | AN ≥ 5000 |  |
| ≥ 0.9% | AC ≥ 2, AN ≥ 150 |  |
| <0.03% | AN>2000 | PM2 |
| Other situations | | Not Evidence |

1. When PM2 is the sole pathogenic evidence, and there exists a sufficient body of benign evidence to establish a benign or suspected benign classification, PM2 evidence shall no longer be deemed as conflicting;
2. Fundamentally, the reference population encompasses the entire population, subpopulations at the continental level, and more localized geographical populations;
3. Principles for Formulating Descriptions of Evidence Items:
   1. Unless otherwise specified, evidence item descriptions should adhere to the general guidelines outlined in the “Guidelines”.
   2. Descriptions should prioritize data from population databases with recorded population information. When providing descriptions, databases with documented population records should be given precedence over those lacking such information.
   3. When creating descriptive statements, it is recommended to follow the sequence of 1000G, ExAC, and gnomAD.
   4. In accordance with the acquired evidence item, priority should be given to describing the population data that support the evidence item. For instance, if the BA1 evidence is based on allele frequency in the EAS population from the gnomAD database, this record should take precedence.
   5. Building upon the preceding clause, when multiple population datasets align with the evidence obtained, it is essential to specify the corresponding population in each database. The order of priority should be as follows: total population, continental-level subpopulations, and geographical populations. In cases where multiple populations at the same level meet the criteria, the population with the highest AF should be selected first.
   6. When creating descriptions, include the AF of the population (in decimal form, rounded to four significant figures) and follow this with the corresponding allele count (AC) and allele number (AN) in parentheses. For example, based on ExAC and gnomAD data, the AF for the NFE population is 0.00001625 (1/61546) and 0.000008891 (1/112478), respectively.
   7. If there is no record in any of the population databases, provide the following description: 'There are no records in the 1000G, ExAC, and gnomAD databases.'"
      1. Variant type analysis (PVS1/PS1/PM5/PP5/BP3)
4. For loss-of-function (LOF) mutations, which include frameshift mutations, nonsense mutations, canonical splice site mutations (IVS ± 1, ± 2), initiation mutations, G>non-G substitutions at the last base of exons, and single or multi-exon deletions, the following considerations should be taken into account for PVS1 evidence.
   1. Careful interpretation is required for LOF mutations near the 3' end of the gene. Nonsense mutations or frameshift mutations occurring after the 1855th amino acid of BRCA1 and 3309th amino acid of BRCA2 do not provide evidence for PVS1.
   2. Some mutations at canonical splicing sites (as indicated in the table below) may potentially result in in-frame deletions or insertions, while certain canonical splicing mutations have been demonstrated to produce naturally occurring in-frame RNA isoforms that could rescue the functionality of *BRCA1* and *BRCA2* genes. Evidence for PVS1 cannot be provided at these sites.

| Gene | Alternative Splicing Event | Mutation Site |
| --- | --- | --- |
| *BRCA1* | △ 8p | c. 442-1, c.442-2 |
|  | △ 9, 10 | c. 548-1, c.548-2, c.593 to non G, c.593+1, c.593+2, c.594-1, c.594-2, c.670 to non G, c.670+1, c.670+2 |
|  | △ 13p | c. 4186-1, c.4186-2 |
|  | △ 14p | c. 4358-1, c.4358-2 |
| *BRCA2* | △ 12p | c. 6842-1, c.6842-2, c.6937 to non G, c.6937+1, c.6937+2 |

- 1. In cases where the first six bases of the intron do not conform to the GTRRGT pattern, a G>non-G substitution at the last base of the exon is more likely to result in abnormal splicing. Conversely, when the first six bases of the intron are GTRRGT, these sites cannot be directly classified as loss-of-function (LOF) variations to support PVS1 evidence (refer to Appendix D).
  2. If a frameshift mutation occurs towards the end of the gene, leading to an extended protein translation, PM4 evidence should be provided.

1. For Missense mutations, the following criteria are applied:
   1. At the same amino acid position, if there is a confirmed pathogenic missense variant with the same amino acid change but a different nucleotide change compared to the variant under classification (splicing-induced pathogenicity should be excluded), PS1 evidence should be assigned.
   2. At the same amino acid position, if there is a confirmed pathogenic missense variant with a different amino acid change (splicing-induced pathogenicity should be excluded), and the amino acid change level of the variant being classified is equal to or higher than that of the known pathogenic missense variant, PM5 evidence is assigned. Amino acid change levels are scored using the Grantham Distance algorithm (PMID: 4843792).
2. For in-frame insertions/deletions, the following criteria are applied:
   1. Unless there are specific circumstances, PM4 evidence can be assigned.
   2. If the mutation occurs in a repetitive region (comprising at least 5 repeating units), and the inserted or missing sequence corresponds to one or multiple repeating units, and the region is functionally uncharacterized, BP3 evidence is assigned.
   3. Additionally, some mutations result in the loss of a stop codon, such as point mutations and deletion mutations occurring in the stop codon. These mutations lead to extended protein translation and should be considered as PM4 evidence.
3. Description of Variant Types:
   1. All variants must include descriptions of their corresponding protein changes, such as:
      1. This mutation is a nonsense mutation, resulting in a substitution from amino acid X to a stop codon at amino acid XX of the gene-coding protein.
      2. This mutation is a frameshift mutation, causing a substitution from amino acid X to amino acid X at amino acid XX of the gene-coding protein and premature translation termination at position XXX.
      3. This mutation is a missense mutation, resulting in a substitution from amino acid X to amino acid X at amino acid XX of the gene-coding protein.
      4. This mutation is a canonical splicing mutation.
   2. For variants providing PVS1 evidence, potential functional effects need to be described:
      1. Frameshift mutation/nonsense mutation may lead to protein dysfunction or inactivation.
      2. Canonical splice site mutation may result in abnormal splicing.
      3. G>non-G at the end of exon: The mutation occurs at the end of an exon, and the downstream 6bp intron sequence is not GTRRGT, potentially causing abnormal splicing.
   3. For mutations occurring at the end of a gene that cannot provide PVS1 evidence, explanations must be provided, such as:
      1. Frameshift mutation/nonsense mutation: Termination occurs after amino acid 1855 of the *BRCA1* gene.
   4. For non LOF mutations, the reasons for obtaining evidence must also be described:
      1. Frameshift mutation/loss of stop codon: Gene XX encodes XX amino acids in its full length, and termination occurs after amino acid XX, resulting in a protein length extension variation.
      2. Mutations involving changes in protein length (in-frame insertion/deletion).
      3. In-frame insertion/deletion refers to an in-frame insertion/deletion occurring in an uncharacterized functional repetitive region.
      4. Missense mutation: This missense mutation shares the same amino acid position as the known pathogenic mutation XXX.
      5. Missense mutation: The missense mutation is identical to the known pathogenic mutation c.xxx p.XXX in terms of amino acid change but has a different nucleotide change." (frameshift mutation /loss of stop codon) XX gene encodes XX amino acids in its full length, and the termination occurs after the XX amino acid, which is a variation of protein length extension.
      6. Public Database Query (BP6/PP5)
4. Retrieve relevant evidence by querying the ClinVar and BRCA Exchange databases:
   1. In cases where the conclusions from both databases conflict, one indicating benign/suspected benign and the other indicating pathogenic/likely pathogenic, no evidence is assigned.
   2. If at least one of the two databases contains records with a pathogenic/suspected pathogenic conclusion, PP5 evidence is assigned.
   3. If at least one of the two databases contains records with a benign/likely benign conclusion, BP6 evidence is assigned.
   4. Please note that in the aforementioned scenarios, ClinVar's conclusions are accepted only if they have a minimum reliability rating of 2 stars; otherwise, they are not considered.
5. ClinVar database website: <https://www.ncbi.nlm.nih.gov/clinvar/>
6. BRCA Exchange database website: <https://brcaexchange.org/>
7. When documenting the evidence, the results obtained from the public database queries must be accurately reported:
   1. In addition to specifying the recorded conclusion, the mutation ID from the ClinVar database and the reliability of that conclusion should be provided.
   2. If no results are obtained from the query, this should also be clearly stated, such as “BRCA Exchange and ClinVar databases have no records”.
      1. Literature Search
8. Literature search constitutes a crucial component of mutation classification interpretation. Various types of evidence outlined in the guidelines can be sourced from literature reports, encompassing but not limited to functional data, co-segregation data, allele data, population data, and emerging data. It is imperative to consolidate all available literature data and corresponding evidence.
9. Various methods can be employed to conduct literature searches:
   1. Certain public databases, such as ClinVar, serve as reliable sources of literature and should be prioritized for searches.
   2. Literature can also be retrieved through search engines, and comprehensive querying is essential, accounting for variations in different naming conventions.
   3. It should be acknowledged that variations in the usage of transcripts might lead to disparities in variation descriptions across different literature sources. Additionally, due to the evolution of variation naming conventions, discrepancies in exon numbering and variation naming and positioning (e.g., CDS location) may exist in early literature. It is imperative to exercise diligence in such cases.
10. Functional Data (BS3/PS3)
    1. In vitro functional testing constitutes a pivotal method for validating mutated function. Mutations confirmed to impair or deactivate function can provide PS3 evidence, whereas mutations confirmed to result in functional normalcy or similarity to wild-type mutations can provide BS3 evidence.
    2. HDR (homology directed recombination) experiments can directly assess the homologous recombination repair function of *BRCA1*/*2* mutants and currently represent a relatively reliable method for functional verification of *BRCA1*/*2* mutations.
    3. mRNA experiments serve as the primary means of determining the splicing impact of variations, particularly synonymous mutations and non-coding region mutations. Two common methods are employed:
       1. The first involves mini-gene experiments, a relatively established approach in the field, with results considered as evidence.
       2. The second entails the analysis of RNA samples extracted from variant carriers, with the conclusions from this method also serving as clear evidence. It is imperative to ascertain whether the experiment accounts for NMD (nonsense-mediated decay) mechanisms.
       3. Both experiments should consider the proportion of abnormal transcripts.
    4. In cases of conflicting experimental outcomes, a thorough analysis of the situation leading to the conflict is necessary. If two conflicting experiments validate different aspects of gene function, corresponding evidence can still be considered based on core function verification results. Otherwise, no evidence is allocated.
11. Case-Control Population Statistics/Case Report Data (PS4)
    1. When comprehensive evidence cannot be obtained through population database queries, literature searches serve as a crucial means of accessing population data. In principle, this should involve surveys and statistical studies encompassing large and medium-sized populations.
    2. If statistical data demonstrate that the AF of the variation in the patient population significantly exceeds that in the general population, PS4 evidence is allocated. It is imperative to ensure that the difference is statistically significant. The OR (Odds Ratio) value can be employed to quantify this difference. If the OR value exceeds 5 and the CI (Confidence Interval) is greater than 1, the criteria for PS4 evidence are met. The OR value can be calculated online (http://www.hutchon.net/ConfidORselect.htm).
    3. In principle, PS4 evidence cannot be used in conjunction with BA1/BS1 evidence. If large-scale population statistics can provide PS4 evidence, careful analysis is needed to determine whether the mutation constitutes a founder mutation within a specific population. If multiple research reports corroborate that the mutation is indeed a founder mutation, PS4 evidence is favored over BA1 and BS1 evidence.
12. Co-Segregation Data (BS4/PP1)
    1. When reports indicate co-segregation with diseases within families, PP1 evidence may be considered. Appropriately, upgrades can be applied based on the reported number of segregated meioses:
       1. If the variant segregates in 3-4 meioses in at least one family, PP1 evidence can be applied.
       2. ii. If the variant segregates in 5-6 meioses in at least one family, PP1_Moderate evidence may be applied.
       3. iii. If the variant segregates in ≥ 7 meioses in at least two families, PP1_Strong evidence may be applied.
    2. If reports demonstrate no co-segregation between the mutation and the diseases, BS4 evidence may be considered:
       1. At least two patients within the reported pedigree clearly do not carry this variant.
       2. At least three healthy individuals carry the mutation, and it is confirmed that their ages exceed the typical age range for disease onset.
13. De Novo Data (PS2/PM6)
    1. If reports indicate that the variant has been detected solely in the proband, with no detection in the parents, and the proband lacks a family history:
       1. If the biological relationship between the proband and patients is confirmed, PS2 evidence is provided.
       2. If the biological relationship between the proband and the patient remains unverified, PM6 evidence is provided.
14. Allelic Data (BP2)
    1. If clear reports suggest that the variant under evaluation coexists with known pathogenic/likely pathogenic variants within a single patient (e.g., breast cancer, ovarian cancer, etc.) or a single healthy individual, BP2 evidence may be considered, contingent upon the following scenarios:
       1. The evaluated variant is observed in trans (in trans configuration) with the known pathogenic/likely pathogenic variant (phase confirmed).
       2. In cases where three or more instances involve the variant along with a pathogenic or likely pathogenic variant (phase unknown), BP2 evidence may be considered. In this context, the variant must be observed in conjunction with at least two different pathogenic/likely pathogenic variants.
15. Other (Phenotypic) Data (PP4)
    1. PP4 serves as phenotypic evidence and should be based on at least two case reports of patients with the mutation, where the patient's phenotype or family history demonstrates a high degree of consistency with the clinical characteristics of gene-related diseases.
    2. In accordance with the recommendations outlined in the NCCN Guidelines (NCCN Genetic/Family High-Risk Assessment: Breast, Ovarian, and Pancreatic), patients can be considered to exhibit the clinical characteristics of *BRCA1*/*2* genetic disorders under the following conditions:
       1. Diagnosis of breast cancer with an onset age of less than 50 years old; or
       2. Diagnosis of triple-negative breast cancer or multiple primary breast cancers; or
       3. Diagnosis of male breast cancer; or
       4. The patient has breast cancer and is of Jewish origin; or
       5. Diagnosis of ovarian epithelial cancer (including fallopian tube cancer or peritoneal cancer); or
       6. Diagnosis of exocrine pancreatic cancer or neuroendocrine pancreatic cancer; or
       7. Diagnosis of metastatic prostate cancer or classification within a high-risk/ultra high-risk group as per the NCCN Prostate Cancer Guidelines for prostate cancer risk assessment.
    3. If the phenotype aligns with the specified criteria, it is also essential to confirm the absence of other genetic pathogenic factors. Various genes, including *ATM*, *BARD1*, *CDKN2A*, *PALB2*, *TP53*, *CDH1*, among others, are known to contribute to these genetic diseases. Different diseases involve distinct genetic genes, and specific NCCN guidelines can be referenced.
    4. PP4 evidence cannot be used concurrently with PS4 evidence. When employing this evidence, it is crucial to ensure that the incidence of mutations in healthy populations is exceedingly low, meeting PM2 evidence requirements.
16. Principles of Documentary Evidence Description
    1. All literature sources must be referenced within parentheses in the evidence description, with priority given to the use of PMID. In instances where no PMID is available, specific numbers such as DOI or website URLs can be employed.
    2. For functional data (BS3/PS3), it is mandatory to delineate the specific experimental methodology and the final conclusions. If conclusions are drawn from key data indicators, these must also be highlighted. In cases where abnormal splicing is confirmed through mRNA analysis experiments, apart from presenting the conclusion, specific splicing events should be described, including the proportion of abnormal transcripts, specific deletions or insertions, and potential protein outcomes (if mentioned in the literature).
    3. For case-control data (PS4), the description should encompass details about the source and size of the surveyed populations (patient group and control group) involved in the study. Key data indicators that support the conclusions, such as p-values, OR (Odds Ratio) values, and corresponding CI (Confidence Interval) values, must be noted. If the study unequivocally establishes the variation as a founder mutation, this should also be clearly indicated. In cases where a public population database serves as the control group, the database version and pertinent population information should be explicitly recorded.
    4. When presenting phenotypic data (PP4), it is imperative to specify whether the symptoms of the case meet the diagnostic criteria for the associated disease or to provide a clear diagnosis conclusion for the case.
    5. For BS4 evidence, the total number of reported cases should be stated, and the source of the literature from which this information is derived must be indicated.
    6. In co-segregation data (PP1), eligible families should be annotated with the number of cases (meioses). If it is established that no co-segregation occurs, the number and circumstances of individuals not conforming to the co-segregation rule should also be included.
    7. In the context of newly released data (PS2/PM6), it is important to indicate whether the biological relationship between parents and the proband is definitively established.
    8. In the case of allele data (BP2), aside from noting the reported number of individuals and diseases (which can also include healthy individuals), the name and classification of another variant, as well as the phase of the two variants, should be explicitly detailed.
       1. Computational Prediction(BP4/BP7/PP3)
17. REVEL, a comprehensive calculation and prediction software, is utilized for functional prediction. In the absence of REVEL results, BayesDel is employed. The respective thresholds and conclusions are outlined in the table below, with threshold determination primarily guided by ClinGen BP4/PP3 evidence recommendations.

| **Software** | **Threshold** | **Classification** |
| --- | --- | --- |
| REVEL | ≥ 0.644 | Disease causing |
|  | (0.290~0.644) | Intermediate |
|  | ≤ 0.290 | Neutral |
| BayesDel | ≥ 0.13 | Disease causing |
|  | (-0.18~0.13) | Intermediate |
|  | ≤ -0.18 | Neutral |

1. For missense mutations, if they are predicted to be disease-causing or to affect splicing, PP3 evidence may be assigned. If they are predicted to be neutral and have no splicing impact, BP4 evidence may be assigned.
2. The comprehensive splicing prediction software, SpliceAI, is universally employed to predict the potential splicing effects:
   1. Predicted scores ≥ 0.5 indicate a predicted splicing effect.
   2. Predicted scores less than 0.5 suggest no predicted splicing effect.
3. For synonymous mutations or intron mutations, splicing prediction suffices. If the prediction indicates a splicing impact, PP3 evidence is assigned; otherwise, BP4 evidence is assigned.
4. In the case of synonymous mutations, if splicing prediction confirms no splicing impact and conservative prediction indicates a non-conservative region, BP7 evidence may be assigned. Conservative prediction presently relies on the PhastCons and PhyloP software for assessment. BP7 evidence is warranted only when both PhastCons scores are <1 and PhyloP scores are <0.1. Otherwise, no BP7 evidence is provided.
5. Alternative Prediction Software: If none of the aforementioned tools are available, other pertinent prediction software may be considered (a selection of prediction software is listed in Appendix E). As a general principle, when predictions from three or more software tools align, corresponding evidence may be provided.
6. Evidence Description: A clear description of the prediction conclusions from the respective software is sufficient:
   1. Based on the prediction from the bioinformatics software REVEL, the variant is deemed neutral/disease-causing/intermediate.
   2. Based on the prediction from the SpliceAI software, it is projected that this variant will not affect splicing/may induce splicing abnormalities.
   3. According to the predictions from sequence conservation prediction software PhastCons46 and PhyloP46, it is determined that the sequence at the mutation position is non-conservative.
   4. If applicable, the outcomes of conservative predictions are noted.
   5. If the mutation has already garnered other predictive evidence, including PVS1, PM4, and BP3, software prediction results may not be provided.
      1. Explanation of Conflicts and Exclusions Between Evidences
7. Computer prediction evidence cannot be simultaneously used, except for BP4+BP7.
8. Population data evidence cannot be simultaneously used.
9. PS4 and PP4 evidence cannot be simultaneously used.
10. When PM2 exists alone and other benign evidence is sufficient to determine benign or likely benign, PM2 is not considered as conflicting evidence.
    - 1. Principles for Writing Variant Interpretation Content:
11. Essential variant description, encompassing the type of variation and resultant amino acid changes.
12. The description is segmented into four sections: basic description, database records, literature records, and computer prediction results. Database records comprise two modules: population data and public database records (ClinVar, BRCA Exchange). Literature records encompass five modules: functional data, co-segregation data, new data, allele data, and other data.
13. Periods separate the four sections, and, except for the literature records section, semicolons demarcate different modules within each section. In the literature records section, periods separate different modules, and semicolons divide information from distinct literature sources within the same module.
14. The description sequence adheres to the order of basic description, population data, public database records, functional data, co-segregation data, new data, allele data, other data, and the module order for calculating prediction data. For instance:
    1. This variant is a missense mutation, resulting in the substitution of arginine with glutamine at amino acid XX of the encoded protein. The allele frequency of the variant is 0.000231 (2/8652) in the ExAC database EAS population, 0.000026 (3/113756) in the gnomADe database NFE population, with no records in the 1000G and gnomADg databases. The variant is reported as pathogenic in the ClinVar database (ID=xxxx, reliability is Three-stars), with no record in the BRCA Exchange database. Results from in vitro protein functional experiments indicate that this mutation impairs protein function (PMID: xxxx, PMID: xxxx). According to literature reports, this mutation was reported as a de novo mutation (PMID: xxxx) in one proband, but the paternity or maternity is not confirmed; In another case report, the variant was identified as de novo (PMID: xxxx) in one proband with confirmed paternity and maternity. Based on the prediction from the bioinformatics software REVEL, the mutation is considered disease-causing.
15. For LOF or in-frame insertion/deletion, the impact on protein function should be described before population data, separated by a period at the end. For example:
    1. This variant constitutes a frameshift mutation, resulting in the substitution of isoleucine with tyrosine at position xxx and a termination at position xxx of the gene-encoded protein. This change may lead to functional impairment or protein inactivation. The allele frequency of this variant in the SAS population of the gnomADe database is 0.0001116 (3/26880), with no recorded instances in the 1000G, ExAC, or gnomADg databases. Notably, this variant is reported as pathogenic in the ClinVar database (ID=xxxx, with a reliability rating of Three-stars). No records of this variant exist in the BRCA Exchange database. A Population Cohort study reveals a significantly higher prevalence of this variant in breast cancer patients compared to controls (PMID: xxxx).
16. During content interpretation, meticulous attention should be directed towards the precise, concise, and semantically coherent use of language. Efforts should be made to circumvent redundancy and cumbersome verbiage.

**Appendix**

Appendix A Evidence Classification and Grading in ACMG Guidelines

Appendix B Standards for Utilizing Evidence in ACMG Guidelines

Appendix C Criteria for Joint Evaluation of Evidence in ACMG Guidelines

Appendix D Table for Assessing Loss-of-Function Variations at Exon Terminals

Appendix E Common Bioinformatics Prediction Software

Appendix A Evidence Classification and Grading in ACMG Guidelines

| **Evidence category** | **Benign** | | | **Pathogenicity** | | | |
| --- | --- | --- | --- | --- | --- | --- | --- |
|  | Alone (BA) | Strong (BS) | Supporting (BP) | Supporting (PP) | Moderate (PM) | Strong (PS) | Very Strong (PVS) |
| Population data | BA1 | BS1; BS2 |  |  | PM2 | PS4 |  |
| Calculation predicted data |  |  | BP1; BP3; BP4; BP7 | PP3 | PM4; PM5 | PS1 | PVS1 |
| Functional data |  | BS3 |  | PP2 | PM1 | PS3 |  |
| Co separated data |  | BS4 |  | PP1 |  |  |  |
| Newly issued data |  |  |  |  | PM6 | PS2 |  |
| Allelic data |  |  | BP2 |  | PM3 |  |  |
| Other databases |  |  | BP6 | PP5 |  |  |  |
| Other data |  |  | BP5 | PP4 |  |  |  |

Note: The evidence in gray font does not apply to the *BRCA1*/*2* gene. Please refer to the main text for specific descriptions.

Appendix B Standards for Utilizing Evidence in ACMG Guidelines

| **Evidence** | **explain** |
| --- | --- |
| PVS1 | Null variant (nonsense, frameshift, canonical +/−1 or 2 splice sites, initiation codon, single or multi-exon deletion) in a gene where loss of function (LOF) is a known mechanism of disease |
| PS1 | Same amino acid change as a previously established pathogenic variant regardless of nucleotide change |
| PS2 | *De novo* (both maternity and paternity confirmed) in a patient with the disease and no family history |
| PS3 | Well-established in vitro or in vivo functional studies supportive of a damaging effect on the gene or gene product |
| PS4 | The prevalence of the variant in affected individuals is significantly increased compared to the prevalence in controls |
| PM1 | Located in a mutational hot spot and/or critical and well-established functional domain (e.g. active site of an enzyme) without benign variation. This evidence is not applicable to *BRCA1*/*2* gene. |
| PM2 | Absent from controls (or at extremely low frequency if recessive) in Exome Sequencing Project, 1000 Genomes or ExAC |
| PM3 | For recessive disorders, detected in trans with a pathogenic variant, and this evidence does not apply to the BRCA1/2 gene |
| PM4 | Protein length changes due to in-frame deletions/insertions in a non-repeat region or stop-loss variants |
| PM5 | Novel missense change at an amino acid residue where a different missense change determined to be pathogenic has been seen before |
| PM6 | Assumed de novo, but without confirmation of paternity and maternity |
| PP1 | Co-segregation with disease in multiple affected family members in a gene definitively known to cause the disease |
| PP2 | Missense variant in a gene that has a low rate of benign missense variation and where missense variants are a common mechanism of disease |
| PP3 | Multiple lines of computational evidence support a deleterious effect on the gene or gene product (conservation, evolutionary, splicing impact, etc) |
| PP4 | Patient’s phenotype or family history is highly specific for a disease with a single genetic etiology |
| PP5 | Reputable source recently reports variant as pathogenic but the evidence is not available to the laboratory to perform an independent evaluation |
| BA1 | Allele frequency is above 1% in Exome Sequencing Project, 1000 Genomes,or ExAC |
| BS1 | Allele frequency is greater than expected for disorder |
| BS2 | Observed in a healthy adult individual for a recessive (homozygous), dominant (heterozygous), or X-linked (hemizygous) disorder with full penetrance expected at an early age, this evidence does not apply to the BRCA1/2 gene |
| BS3 | Well-established in vitro or in vivo functional studies shows no damaging effect on protein function or splicing |
| BS4 | Lack of segregation in affected members of a family |
| BP1 | Missense variant in a gene for which primarily truncating variants are known to cause disease |
| BP2 | Observed in trans with a pathogenic variant for a fully penetrant dominant gene/disorder; or observed in cis with a pathogenic variant in any inheritance pattern |
| BP3 | In-frame deletions/insertions in a repetitive region without a known function |
| BP4 | Multiple lines of computational evidence suggest no impact on gene or gene product (conservation, evolutionary, splicing impact, etc) |
| BP5 | Variant found in a case with an alternate molecular basis for disease. This evidence does not apply to the *BRCA1*/*2* gene |
| BP6 | Reputable source recently reports variant as benign but the evidence is not available to the laboratory to perform an independent evaluation |
| BP7 | A synonymous (silent) variant for which splicing prediction algorithms predict no impact to the splice consensus sequence nor the creation of a new splice site AND the nucleotide is not highly conserved |

Appendix C Criteria for Joint Evaluation of Evidence in ACMG Guidelines

| Clinical significance classification | Rules for Combining Criteria | |
| --- | --- | --- |
| 5-Pathogenic | (1) 1 PVS+≥ 1 PS | (2) 1 PVS+≥ 2 PM |
|  | (3) 1 PVS+1 PM+1 PP | (4) 1 PVS+≥ 2 PP |
|  | (5) ≥ 2 PS | (6) 1 PS+≥ 3 PM |
|  | (7) 1 PS+2 PM+≥ 2 PP | (8) 1 PS+1 PM+≥ 4 PP |
| 4- Suspected pathogenic | (1) 1 PVS+1 PM | (2) 1 PS+1-2 PM |
|  | (3) 1 PS+≥ 2 PP | (4) ≥ 3 PM |
|  | (5) 2 PM+≥ 2 PP | (6) 1 PM+≥ 4 PP |
| 1- Benign | (1) 1 BA | (2) ≥ 2 BS |
| 2- Suspected benign | (1) 1 BS+1 BP | (2) ≥ 2 BP |
| 3- Ambiguous meaning | (1) Does not meet the above criteria | (2) Contradiction between benign and pathogenic criteria |

Appendix D Table for Assessing Loss-of-Function Variations at Exon Terminals

| Serial number | BRCA1 gene | | | BRCA2 gene | | |
| --- | --- | --- | --- | --- | --- | --- |
|  | First 6 bases of Intron | Donor GTRRGT | Is it LOF | First 6 bases of Intron | Donor GTRRGT | Is it LOF |
| Exon 1 | GTAGTA | N | yes | GTTAGT | Y | no |
| Exon 2 | GTAAGT | Y | no | GTATTG | N | yes |
| Exon 3 | GTAAGT | Y | no | GTAAGT | Y | no |
| Exon 4 | - | - | - | GTATGA | N | yes |
| Exon 5 | GTATAT | N | yes | GTATGA | N | yes |
| Exon 6 | GTAAGT | Y | no | GTAAAT | N | yes |
| Exon 7 | GTAAAA | N | yes | GTAATA | N | yes |
| Exon 8 | GTAAGG | N | yes | GTAAGT | Y | no |
| Exon 9 | GTGAGT | Y | no | GTAAGT | Y | no |
| Exon 10 | GTAATG | N | yes | GTACCT | N | yes |
| Exon 11 | GTATTG | N | yes | GTAAGT | Y | no |
| Exon 12 | GTAAAA | N | yes | GTAAAA | N | yes |
| Exon 13 | GTGTGT | N | yes | GTAAGA | N | yes |
| Exon 14 | GTAAGA | N | yes | GTATTG | N | yes |
| Exon 15 | GTAATA | N | yes | GTATGT | N | yes |
| Exon 16 | GTGAGT | Y | no | GTACTC | N | yes |
| Exon 17 | GTATAC | N | yes | GCAAGT | N | yes |
| Exon 18 | GTAAGT | Y | no | GTAAAT | N | yes |
| Exon 19 | GTAAGT | Y | no | GTATGA | N | yes |
| Exon 20 | GTAAAG | N | yes | GTAAAA | N | yes |
| Exon 21 | GTAAGA | N | yes | GTGAGA | N | yes |
| Exon 22 | GTAAGT | Y | no | GTAAGT | Y | no |
| Exon 23 | GTAAGG | N | yes | GTACAA | N | yes |
| Exon 24 | - | - | - | GTAATG | N | yes |
| Exon 25 | - | - | - | GTAAGG | N | yes |
| Exon 26 | - | - | - | GTAAGT | Y | no |

Appendix E Common Bioinformatics Prediction Software

| **Prediction type** | **Prediction software** | **Website** |
| --- | --- | --- |
| Missense prediction | REVEL | http://grch37.ensembl.org/Homo_sapiens/Tools/VEP |
|  | BayesDel | http://grch37.ensembl.org/Homo_sapiens/Tools/VEP |
|  | MutationTaster | https://www.mutationtaster.org/ |
|  | PolyPhen-2 | http://genetics.bwh.harvard.edu/pph2/ |
|  | Provean | http://grch37.ensembl.org/Homo_sapiens/Tools/VEP |
|  | SIFT | http://grch37.ensembl.org/Homo_sapiens/Tools/VEP |
| Splicing prediction | SpliceAI | http://grch37.ensembl.org/Homo_sapiens/Tools/VEP |
|  | FSPLICE | http://www.softberry.com/berry.phtml?topic=fsplice&group=programs&subgroup=gfind |
|  | MaxEntScan | http://hollywood.mit.edu/burgelab/maxent/Xmaxentscan_scoreseq.html |
|  | NetGene2 | https://services.healthtech.dtu.dk/service.php?NetGene2 -2.42 |
|  | NNsplice | https://www.fruitfly.org/seq_tools/splice.html |
| Nucleic acid conservation prediction | PhastCons | Http://compgen. bscb. cornell. edu/phast/ |
|  | PhyloP | Http://compgen. bscb. cornell. edu/phast/ |
